# Attitudes Towards End-of-Life Care Among Nursing Students: A Cross-Sectional Descriptive Study in a Southern European Undergraduate Nursing Program

**DOI:** 10.3390/nursrep16070233

**Published:** 2026-07-03

**Authors:** Eduardo Sánchez-Sánchez, Cristina Sánchez-Fernández, Nerea Listán-Barranco, Carmen Rocha-Domínguez, Jara Díaz-Jiménez, Nuria Trujillo-Garrido

**Affiliations:** 1 Faculty of Nursing, Department of Nursing and Physiotherapy, Venus Street, 11207 Cádiz, Spain; eduardo.sanchez@uca.es (E.S.-S.); cristina.sanchezfernandez@uca.es (C.S.-F.); nerealb99@gmail.com (N.L.-B.); carmen.rochadominguez@alum.uca.es (C.R.-D.); 2 Institute of Research and Innovation in Biomedical Sciences of the Province of Cádiz (INIBICA), Hospital Universitario Puerta del Mar, Av. Ana de Viya 21, 11009 Cádiz, Spain; 3 Department of Didactics of Physical, Plastic and Musical Education, Faculty of Education Sciences, University of Cádiz, 11519, Cádiz, Spain; jara.diaz@uca.es

**Keywords:** end-of-life care, nursing students, attitudes, nursing education

## Abstract

**Background/Objectives:** Attitudes toward end-of-life care (EOLC) are a key component of nursing practice. This study aimed to assess nursing students’ attitudes toward EOLC and their perceived preparedness to manage end-of-life situations. **Methods:** A cross-sectional descriptive study was conducted with 593 undergraduate nursing students from a public Spanish university. Data were collected using an online questionnaire, including the validated Spanish version of the Frommelt Attitude Toward Care of the Dying Scale (FATCOD-S). Descriptive and inferential analyses were performed. **Results:** The median reverse-coded FATCOD-S total score was 125.0 (IQR 119.0–131.0), and 99.7% of students were classified as having positive or very positive attitudes when the descriptive cut-offs were applied. In the exploratory adjusted model, fourth-year status and previous EOLC training were associated with higher FATCOD-S total scores. However, 59.5% of respondents reported feeling unprepared to provide EOLC, and 59.0% perceived EOLC as a significant source of stress for nurses. Additionally, 62.0% of students with positive attitudes reported not feeling prepared to provide such care. Responses related to emotional involvement, communication about death, and ethical aspects showed greater variability. **Conclusions:** Although most nursing students display favorable attitudes toward EOLC, these coexist with a low perceived level of preparedness, with more than half of students reporting that they do not feel prepared to provide EOLC. Positive attitudes alone may not ensure confidence in clinical practice. Strengthening undergraduate education—particularly in emotional preparation, communication skills, and coping strategies—is essential to better prepare future nurses for the complexities of EOLC. These findings should be interpreted in light of the study’s cross-sectional design and single-university setting.

## 1. Introduction

End-of-life care (EOLC) aims to improve the comfort of patients not only in the physical domain, which is associated with a higher quality of life and a reduction in hospitalizations [1], but also in psychosocial and spiritual dimensions [2]. These areas of care have evolved in response to societal changes, the emergence of new treatments, diseases, healthcare technologies, and other advancements. Consequently, new concepts, legislation, and evidence-based approaches have emerged with the shared objective of ensuring that patients receive dignified care at the end of life [3,4,5].

In this context, nurses play a fundamental role in EOLC [6], as they often provide continuous care to patients over extended periods. Their work focuses on offering dignified and respectful treatment to both the patient and their family, encompassing a holistic approach that addresses physical, psychological, social, and spiritual dimensions, with particular attention to the dying process [3,4]. This work can be complex and emotionally demanding, which may increase stress and anxiety among the nurses providing this type of care [7,8]. The emotions they experience include helplessness, uncertainty, guilt, frustration, and anger, which may contribute to the development of emotional exhaustion, depersonalization, and a decreased perception of their own professional competence [9,10,11]. These factors not only affect their well-being but may also negatively impact the quality of care provided, potentially impairing the delivery of EOLC [12]. Moreover, this type of care is essential for patients of all ages, from infants to adolescents and young adults [13]. It is crucial to note that the process of caring for babies, children, and adolescents at the end of life, as well as coping with their death, can be particularly traumatic and emotionally devastating for many nurses, causing profound distress on both a personal and professional level [14].

The Australian College of Critical Care Nurses (ACCCN) developed a statement that includes specific recommendations for nurses in Critical Care Units regarding the provision of EOLC, in response to the widespread perception of insufficient training in this area [15]. Similarly, other healthcare settings, including emergency departments, have adopted measures to provide EOLC, focusing on maximizing patient comfort and managing symptoms in order to prevent unnecessary suffering and preserve dignity [16]. In primary care, an increasing number of terminally ill patients are opting to receive care at home, reflecting a growing trend towards home-based EOLC [17]. Additionally, in the social care sector, nursing homes have also become key settings for the provision of EOLC [18].

In this context, undergraduate nursing education significantly influences the quality of EOLC. Previous research has shown that nursing students and newly graduated nurses often experience difficulties when facing end-of-life situations. These challenges include communicating with dying patients and their families, managing emotional distress associated with death, and feeling insufficiently prepared to provide end-of-life care. Such difficulties may negatively affect confidence, coping abilities, and the quality of care provided, highlighting the importance of adequate undergraduate preparation in this area [19,20,21].

Attitudes towards end-of-life care within undergraduate nursing education are commonly assessed using validated self-report instruments. One of the most widely used measures is the Frommelt Attitude Toward Care of the Dying Scale (FATCOD) (Frommelt, 1991), which has been extensively applied among nursing students across a range of educational and cultural settings. However, previous studies using the FATCOD have reported heterogeneous findings, often influenced by curricular structure, clinical exposure, and sociocultural factors. Accordingly, although some studies have shown that students with previous experience or specific training in the care of dying patients tend to report more positive attitudes towards end-of-life care, findings vary across educational and cultural contexts [22,23,24]. More specifically, in Spain, undergraduate nursing education is structured as a four-year program, in which clinical placements generally start in the second year and dedicated theoretical training in end-of-life care is commonly provided in the third year. This training is typically delivered through lectures, seminars, and case-based learning activities focused on principles of EOLC, symptom management, communication with patients and families, ethical decision-making, grief and bereavement, psychosocial support, and interdisciplinary care.

Nevertheless, evidence from Southern European undergraduate nursing programs remains limited, highlighting the need for further research in comparable educational contexts. Furthermore, differences in curricular organization, timing of clinical placements, and the provision of formal end-of-life care education may influence students’ attitudes towards EOLC. Consequently, findings from other educational and cultural settings cannot be directly extrapolated to Southern European nursing programs. Additional evidence is therefore needed to better understand students’ attitudes within this specific educational context.

Therefore, the aim of the present study was to explore nursing students’ attitudes towards end-of-life care within a Southern European undergraduate nursing program, identifying potential educational gaps with relevance for similar educational settings.

## 2. Materials and Methods

### 2.1. Study Design and Respondents

A cross-sectional descriptive study was conducted using an online questionnaire distributed through a web-based platform among undergraduate nursing students enrolled in a public nursing education program in Spain. The nursing degree program has a duration of four years, and clinical placements begin in the second year. The program combines theoretical training with clinical placements throughout the degree. Students with contraindications for using online questionnaires were excluded.

A convenience sampling method was used. The reference population comprised 1312 undergraduate nursing students enrolled in the nursing centers of the University of Cádiz (Faculty of Nursing Campus Bahía de Algeciras, Faculty of Nursing and Physiotherapy in Cádiz, Faculty of Nursing and Physiotherapy in Jerez, and the Salus Infirmorum University Nursing Centre). The sample-size calculation assumed an expected proportion of 50% (maximum variability), a 95% confidence level (Z = 1.96), and a precision of 3%. After applying finite population correction for *n* = 1312, the minimum required sample was 589 students. The target was increased to approximately 600 students to allow for invalid or incomplete questionnaires. A total of 594 questionnaires were received, and 593 were valid, representing 45.2% of the reference population. Because respondents were recruited through a voluntary convenience sampling strategy, the sample should not be interpreted as probabilistic or fully representative of all nursing students.

### 2.2. Outcome Measures and Instruments

Sociodemographic variables were collected (gender, age, and experience with a loved one who had received EOLC); academic variables (academic year, completion of external academic placements, previous training on EOLC); and additional variables related to students’ perceived preparedness to provide EOLC, perceptions of the nursing role in EOLC, perceived stress associated with this type of care, and the occurrence of ethical conflicts.

To study attitudes towards EOLC, the validated Spanish version of the Frommelt Attitude Toward Care of the Dying Scale (FATCOD-S) was used. This instrument was culturally adapted and psychometrically validated in Spanish nursing students to assess attitudes towards caring for terminally ill patients and their families [25]. The FATCOD-S is a Likert-type scale composed of 30 items, 13 of which are reverse-worded items. Each item is assessed on a 5-point scale, with response options ranging from: strongly disagree (1 point), disagree (2 points), neutral (3 points), agree (4 points), and strongly agree (5 points). Reverse items were recoded prior to analysis so that higher scores consistently reflected more positive attitudes towards EOLC.

The FATCOD-S total score ranges from 30 to 150, with higher scores reflecting more positive attitudes towards EOLC. In the present study, the FATCOD-S was handled primarily as a continuous measure after reverse coding of negatively worded items [25]. For descriptive purposes and to facilitate comparison with previous research, previously published cut-off points were also applied: 30–60 points, very negative attitudes; 61–90 points, negative attitudes; 91–120 points, positive attitudes; and 121–150 points, very positive attitudes [26]. These categories are not universally established thresholds and were therefore interpreted only as descriptive groupings rather than psychometric or diagnostic categories.

The original Frommelt Attitude Toward Care of the Dying (FATCOD) scale was developed as a unidimensional instrument to assess attitudes towards the care of terminally ill patients and their families. The validated Spanish adaptation used in the present study (FATCOD-S) derives from this original instrument. However, subsequent validation studies have identified different factorial structures depending on the population and cultural context.

For the purposes of result presentation and interpretation, six conceptual domains related to relational aspects of EOLC were considered. These domains were used solely to facilitate the interpretation and presentation of the results. They were not treated as independent psychometric subscales, and no confirmatory factor analysis or additional psychometric testing was performed to evaluate this classification in the present sample. Items 3, 5, 7, 8, 13, 14, 15, and 26 were grouped under emotional discomfort in caring for dying patients. Items 4, 16, and 22 referred to nursing care directed towards the patient’s family. Items 2, 6, 11, 27, 28, and 30 were related to nurse communication and information in EOLC. Items 12, 18, and 20 addressed the role of the family as caregivers. Items 1, 9, 10, 17, 21, and 29 were associated with relational aspects of EOLC, while items 19, 23, 24, and 25 referred to patient autonomy in EOLC. Overall, two thirds of the items relate directly to attitudes towards the patient, whereas the remaining third refer to attitudes towards the patient’s family (Supplementary Material).

The Spanish version of the FATCOD-S has previously demonstrated adequate validity and reliability in undergraduate nursing students. In the present study, the FATCOD-S showed good internal consistency (Cronbach’s α = 0.78).

### 2.3. Data Collection

Data collection was conducted in person by a member of the research team during scheduled academic activities. Eligible students were informed about the study aims and invited to participate. Students who agreed to participate were provided with a QR code linking to an anonymous online questionnaire hosted on Google Forms (Google LLC, Mountain View, CA, USA), which they completed electronically using their own devices. Participation was voluntary, no incentives were offered, and students could decline participation without academic consequences. As no identifying information was collected, the characteristics of students who did not participate could not be compared with those of respondents. Duplicate or incomplete responses were screened before analysis.

### 2.4. Data Analysis

Descriptive analyses were conducted for all study variables. Categorical variables were reported as frequencies and percentages. Quantitative variables, including age and FATCOD-S total/domain scores, were reported as medians and interquartile ranges because the Lilliefors (Kolmogorov–Smirnov) test and visual inspection indicated non-normal distributions.

The FATCOD-S total score was calculated by summing the 30 items after reverse coding negatively worded items so that higher scores consistently reflected more positive attitudes towards EOLC. The total score was analyzed as a continuous measure. The four attitude categories based on published cut-offs were used as secondary descriptive groupings and were not treated as universally validated thresholds. Specifically, the 15 negatively worded items identified in the FATCOD-S scoring procedure were reverse-coded before summing the total score.

For bivariate analyses, categorical variables were compared using the chi-square test or Fisher’s exact test when expected cell counts were small. For non-normally distributed quantitative variables, the Mann–Whitney U test or the Kruskal–Wallis test was used according to the number of comparison groups. Item-level FATCOD-S results were treated as ordinal descriptive data and are presented as response frequencies and medians with interquartile ranges; no inferential comparisons were conducted at item level, and therefore Table 2 does not include *p*-values.

To account for potential confounding, an exploratory multivariable linear regression model was performed using the reverse-coded FATCOD-S total score as the dependent variable. Covariates were selected a priori and included age, gender, academic year, previous experience with a loved one receiving EOLC, previous EOLC training, and completion of a clinical placement in an EOLC unit. Regression coefficients (β), 95% confidence intervals (CI), and *p*-values were reported. Robust HC3 standard errors were used. Because of the very small number of respondents who identified as non-binary (*n* = 2), these respondents were retained in descriptive analyses but excluded from the adjusted model to avoid unstable estimates. The model was exploratory and was used to estimate adjusted associations rather than causal effects.

Because several bivariate and domain-level analyses were performed, *p*-values from these analyses were interpreted cautiously. No formal correction for multiple comparisons was applied because these analyses were exploratory rather than confirmatory. The main inferential interpretation was based on the FATCOD-S total score and the adjusted multivariable model.

A significance level or type I error of 0.05 was used. The R statistical program (R Foundation for Statistical Computing, Vienna, Austria) [27] was utilized.

### 2.5. Ethical Considerations

The project followed the ethical principles of the 2013 revision of the Declaration of Helsinki and was reviewed and approved by the Provincial Research Ethics Committee with Medicines (CEIm) of Cádiz on 6 February 2024, with registration number SICEIA-2024-000131. All respondents provided written informed consent to participate in the study and for their data to be published.

## 3. Results

Of the 594 students who completed the questionnaire, one was excluded due to duplicate or incomplete responses, resulting in a final sample of 593 respondents.

### 3.1. Respondents’ Sociodemographic and Academic Characteristics and Experiences Related to EOLC

The median age of the respondents was 21.0 years (IQR 20.0–22.0). Most respondents identified as female (83.98%, *n* = 498), followed by male (15.68%, *n* = 93) and non-binary respondents (0.34%, *n* = 2).

Most respondents were second-year students (36.1%, *n* = 214), while the lowest percentage was observed among final-year students, with 14.2% (*n* = 84).

All respondents (100.00%, *n* = 593) considered EOLC to be important, and 99.66% (*n* = 591) reported that training in this type of care is important.

Overall, 43.68% (*n* = 259) reported having received training in EOLC, although only one in ten considered the training received to be sufficient. In addition, 53.46% (*n* = 317) of respondents believed that nurses are taken into account in the management of EOLC, whereas 59.02% (*n* = 350) considered that providing this type of care generates a significant level of stress among nurses (Table 1).

### 3.2. Attitudes Towards EOLC Measured with the FATCOD Scale

408 students (68.8%) were classified as having very positive attitudes, 183 (30.9%) as having positive attitudes, 1 (0.2%) as having negative attitudes and 1 (0.2%) as having very negative attitudes. Thus, 99.7% (*n* = 591) of respondents were classified as having positive or very positive attitudes towards EOLC. Overall, the item-level responses showed strong support for family-centered care, communication with dying patients and their families, and continued nursing involvement at the end of life, whereas greater uncertainty was observed in items related to emotional involvement, death-related beliefs, and family-professional interactions.

Positive attitudes towards patient- and family-centered care were strongly reflected in several items of the FATCOD-S scale. For example, 89.88% of students strongly agreed that families need emotional support to cope with behavioral changes in dying patients (Item 16), and 85.83% strongly agreed that it is beneficial for dying persons to verbalize their feelings (Item 21). Similarly, 81.45% strongly agreed that nursing care should extend to the family of the dying person (Item 4), and 75.38% considered caring for dying patients to be a worthwhile learning experience (Item 1).

In addition, attitudes suggesting avoidance or withdrawal from EOLC were overwhelmingly rejected. A large majority of students (87.35%) strongly disagreed with the statement that nurses should withdraw their involvement as a patient nears death (Item 17). Likewise, 60.71% strongly disagreed that nurses should avoid discussing death with dying patients (Item 6), indicating broad acceptance of the nurse’s role in communication and support at the end of life.

However, several items related to ethical beliefs, emotional involvement, and communication about death showed more heterogeneous response patterns. Notably, the most frequent response to the statement “Death is not the worst thing that can happen to a person” was the neutral option (31.70%; Item 2). Similarly, the most frequent response to the statement “I would feel uncomfortable talking to a person who is dying about their own death” was also neutral (36.93%; Item 3). Students also showed divided responses regarding fear of forming friendships with dying patients (Item 14; Me = 3.0). Likewise, perceptions of whether family members staying close to the patient interfere with professional care showed a high proportion of neutral responses (44.18%; Item 29) (Table 2).

**Figure 1:**
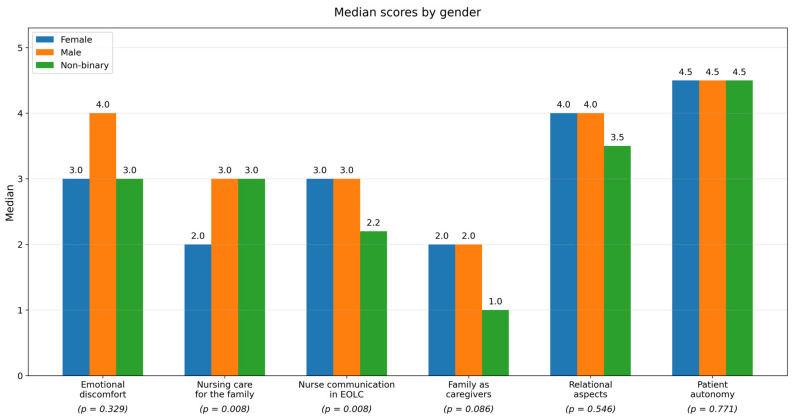
Median scores obtained in the subdimensions of the FATCOD-S scale by gender.

Figure 1 presents the median scores obtained in the different FATCOD-S subdimensions according to gender. Using the reverse-coded FATCOD-S total score, median scores were similar across gender groups: female respondents, 125.0 (IQR 119.0–131.0); male respondents, 125.0 (IQR 114.0–133.0); and non-binary respondents, 127.0 (IQR 126.0–128.0). Differences were not statistically significant (Kruskal–Wallis *p* = 0.789). Because only two respondents self-identified as non-binary, gender-based comparisons should be interpreted with caution. These gender-specific domain-level comparisons should be considered exploratory, and no substantive conclusions regarding non-binary respondents can be drawn from these results. In the subdimension “emotional discomfort in caring for dying patients”, male respondents showed a higher median score (4.0) than female and non-binary respondents (both 3.0), although these differences were not statistically significant (*p* = 0.329). As negatively worded items were reverse-coded before calculating the subdimension scores, higher values indicate lower emotional discomfort when caring for dying patients.

In the subdimension “nursing care directed towards the patient’s family”, a median value of 2.0 was observed among female respondents, whereas male and non-binary respondents presented a value of 3.0. These differences were statistically significant (*p* = 0.008) (Figure 1).

Statistically significant differences were also observed in the subdimension “nurse communication and information in EOLC” (*p* = 0.007), with median values of 3.0 for female and male respondents and 2.2 for non-binary respondents.

These domain-level findings should be interpreted as exploratory because several comparisons were performed and no formal multiplicity correction was applied. Therefore, the *p*-values should not be interpreted as confirmatory evidence on their own; they should be considered together with the observed medians, the direction of the differences, and their potential educational relevance.

Using the reverse-coded FATCOD-S total score, median scores by academic year were 123.0 (IQR 115.0–131.0) in first-year students, 125.0 (IQR 119.0–131.0) in second-year students, 124.0 (IQR 119.0–131.0) in third-year students, and 130.0 (IQR 126.0–137.0) in fourth-year students. Differences were statistically significant (Kruskal–Wallis *p* < 0.001). As these comparisons are unadjusted, they should be interpreted together with the multivariable model.

**Figure 2:**
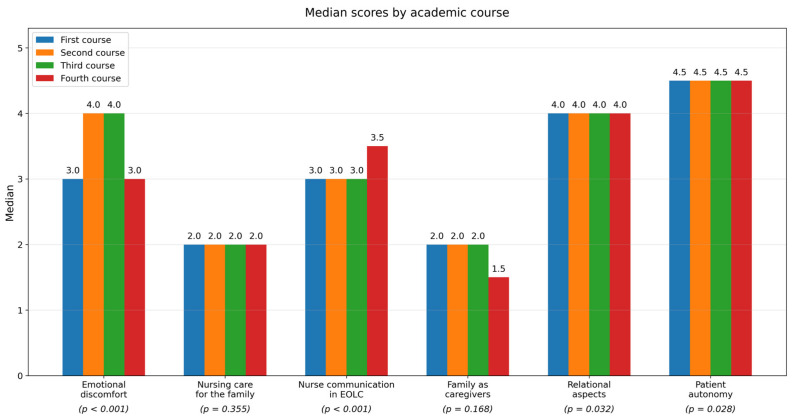
Median scores obtained in the subdimensions of the FATCOD-S scale by academic year.

Median scores across the different subdimensions were generally similar across academic years, although statistically significant differences were observed in several domains of the scale (Figure 2). The most notable differences were found in the subdimension “emotional discomfort in caring for dying patients” (*p* < 0.001), where second- and third-year students showed higher median scores than first- and fourth-year students. Significant differences were also observed in “nursing care directed towards the patient’s family” (*p* = 0.035), “nurse communication and information in EOLC” (*p* < 0.001), “relational aspects of EOLC” (*p* = 0.032), and “patient autonomy in EOLC” (*p* = 0.028). No statistically significant differences were found in the subdimension “role of the family as caregivers” (*p* = 0.168).

These differences should be interpreted cautiously because they derive from exploratory domain-level comparisons and may be influenced by training exposure, clinical placements, and previous personal experience with EOLC.

### 3.3. Exploratory Multivariable Analysis

Because only two respondents were classified in the negative or very negative categories after reverse coding, cross-tabulations by attitude category were not considered analytically informative. Associations between educational or experiential variables and attitudes were therefore examined using the continuous reverse-coded FATCOD-S total score in the adjusted model presented below.

After complete-case adjustment (*n* = 572), previous EOLC training and academic year were associated with the reverse-coded FATCOD-S total score. Compared with first-year students, fourth-year students had higher FATCOD-S total scores (β = 4.83, 95% CI 1.63 to 8.03, *p* = 0.003). Students who had received previous EOLC training also had higher scores than those without training (β = 2.29, 95% CI 0.03 to 4.55, *p* = 0.047). Previous experience with a loved one receiving EOLC (β = 1.54, 95% CI −0.17 to 3.25, *p* = 0.077) and completion of a clinical placement in an EOLC unit (β = 1.87, 95% CI −0.32 to 4.06, *p* = 0.095) showed positive but non-significant associations. Age, male gender, second-year status and third-year status were not statistically significant. The model explained a modest proportion of variance (R^2^ = 0.075; adjusted R^2^ = 0.062), so these findings should be interpreted as exploratory (Table 3).

## 4. Discussion

This study explored nursing students’ attitudes towards EOLC, as well as their perceptions of the training received and their preparedness to face these situations in clinical practice.

The FATCOD-S results indicated generally positive attitudes towards EOLC among nursing students included in this study. In addition, all respondents considered EOLC to be important, and the vast majority stated that all patients have the right to receive it. These findings are relevant because nurses’ attitudes towards death and terminal care may influence the quality of the support they provide in end-of-life situations; in particular, more negative attitudes have been associated with a lower willingness to engage in this type of care [11,28]. In this regard, the favorable attitudes observed in our sample may be associated with a greater willingness to provide EOLC.

One of the most relevant findings of this study is the coexistence of predominantly positive attitudes towards EOLC and a low perceived level of preparedness to deal with such situations. This result may suggest that a positive appraisal of this type of care does not necessarily translate into a sense of confidence in addressing it in clinical practice. However, preparedness was assessed through a single self-reported item and should therefore be interpreted as a subjective perception of readiness rather than as an objective measure of clinical competence. This apparent discrepancy may partly reflect characteristics of the educational context in which students are trained.

In Spain, as in other European countries that follow programs structured according to the Bologna model, undergraduate nursing programs last four academic years, and specific training in EOLC is often concentrated in the later stages of the curriculum. As a result, many students may encounter EOL situations during their early clinical placements before having received structured theoretical or practical training in this area. Early exposure to EOL situations in the absence of sufficient formal preparation may help explain this apparent discrepancy, contributing to students recognizing the importance of this type of care through direct clinical experience, while at the same time limiting their confidence and perceived preparedness due to the lack of structured training and emotional support [21,29,30].

Beyond these educational considerations, greater exposure to end-of-life situations during nursing training may contribute to a more realistic appreciation of the clinical, emotional, ethical, and organizational complexity of EOLC [31,32,33]. In this sense, more direct contact with situations of death and with the suffering of patients and their families may be accompanied by more demanding emotional experiences and a greater awareness of the challenges associated with this type of care, which could influence how students evaluate these situations compared with earlier stages of their training [29,30,31,34]. Although this pattern may be relevant to educational contexts with comparable curricular structures, it should be interpreted cautiously because the sample was drawn from a single university using a convenience sampling strategy.

Taken together, these results may indicate that favorable attitudes alone may not be sufficient to ensure a sense of confidence or perceived competence in providing EOLC, particularly in the absence of adequate training and emotional preparation. From an educational perspective, these findings may have important implications for undergraduate nursing education, particularly in programs with curricular structures similar to that examined in this study. It may therefore be appropriate to address the observed gap between positive attitudes and perceived preparedness through educational programs that place greater emphasis not only on the development of clinical knowledge in EOLC, but also on emotional preparation, communication skills, and coping strategies related to death and the dying process. In this context, teaching approaches such as simulation-based learning, guided reflection, and supervised clinical exposure may facilitate the integration of theoretical knowledge with emotional and experiential learning, thereby helping to improve students’ confidence and perceived preparedness to face EOL situations in clinical practice [35,36,37].

Students’ perceptions of the training received provide further insight into this apparent discrepancy. Continuing with the issue of training, our results indicate that a high proportion of students considered the training they had received to cope with EOLC situations to be insufficient. This perception was observed both among students with positive attitudes towards this type of care and among those with less favorable attitudes, suggesting that it may not be limited to a specific subgroup and may be relatively widespread within the study sample.

In fact, although most respondents considered training in EOLC to be important, only one in ten of the students who had received prior training considered it to be sufficient. In this regard, the literature has shown that knowledge about EOLC directly influences the quality of care, and that insufficient training in this area may constitute a barrier to providing high-quality EOLC [38,39,40]. Thus, when students do not have adequate preparation, their perceived ability to provide high-quality care after graduation may be compromised, which may lead to stress, insecurity, or reluctance when facing such situations, with the consequent risk that patients’ needs and preferences may not be fully addressed [41].

Further insight into this issue is provided by the item-level findings. In particular, some items of the FATCOD-S scale related to emotional involvement with terminally ill patients, the role of family members during the dying process, and clinical decision-making in EOL situations showed more heterogeneous response patterns. This finding may suggest that, although students recognize the value of EOLC, they may experience greater uncertainty when facing the emotionally complex or ethically sensitive aspects of this type of care [20]. In this regard, structured educational interventions aimed at developing emotional competencies and communication skills could help students translate positive attitudes into greater confidence when facing EOL situations in clinical practice. These findings should be interpreted with caution, as the conceptual domains were used solely for descriptive and interpretive purposes and were not evaluated as independent psychometric subscales through confirmatory factor analysis or other psychometric procedures.

Continuing with the ethical dimension of EOLC, it is noteworthy that, although ethical or moral conflicts were reported by a minority of respondents (11.8%), they were observed more frequently among students who displayed positive attitudes towards this type of care. This finding may reflect, among other factors, a greater sensitivity or awareness of the ethical complexity inherent in EOL situations, in which issues related to patient autonomy, suffering, family involvement, and clinical decision-making are involved [42,43].

Furthermore, a considerable proportion of students (60%) believed that EOLC generates high levels of stress among nurses, reflecting the emotional demands associated with caring for patients at this stage. In this regard, some studies have shown that professionals who have not received specific training in EOLC experience higher levels of death-related anxiety and stress compared with those who have received such training, highlighting the importance of incorporating specific educational programs in this area [7,44].

Moreover, the results also indicate that students do not consistently perceive that nurses are taken into account in the management of EOLC. This perception may reflect limited exposure during undergraduate training to organizational aspects and decision-making processes related to this type of care, highlighting the need to integrate into education not only clinical and emotional content but also the professional role of nursing in the planning and management of EOLC [45,46].

Overall, within the context of this study, these results may reinforce the need for undergraduate nursing education to prepare students not only for the clinical aspects of EOLC, but also for its emotional, ethical, organizational, and decision-making dimensions.

Academic year was also associated with differences in attitudes towards EOLC. When attitudes were analyzed according to academic year using the reverse-coded FATCOD-S total score, statistically significant differences were observed across groups, with the highest median score found among fourth-year students. This pattern may reflect the influence of academic progression, curricular exposure, previous training, and increasing contact with clinical practice throughout the nursing program. Previous studies have reported that, as students gain experience and encounter real clinical situations—including contact with seriously ill patients and their families— they may develop greater familiarity and confidence in addressing EOLC [22,24,47]. In this regard, several studies have suggested that undergraduate education in EOLC can contribute to improving students’ attitudes towards death and the provision of EOLC [19,23]. Although previous studies have reported such associations, the present cross-sectional design does not allow us to determine whether the differences observed across academic years are attributable to educational progression itself.

Furthermore, these findings should be interpreted with caution. Exposure to clinical placements, previous personal experience, and previous EOLC training may influence FATCOD-S scores, and these factors may also overlap with academic year. Therefore, the observed differences should be interpreted as associations rather than causal effects. Claims about independent associations should be limited to variables that remained associated in the adjusted model, particularly fourth-year status and previous EOLC training.

This interpretation is further supported by the adjusted analysis. From a methodological perspective, previous EOLC training and clinical exposure may act as potential confounding factors. Approximately one third of respondents had completed placements in units where EOLC was provided, and fewer than half had received previous EOLC training. These variables may shape students’ attitudes and perceived preparedness, and they may also overlap with academic year. Therefore, bivariate differences by academic year should not be interpreted in isolation. In this study, the adjusted analysis provides a more appropriate basis for assessing whether educational stage, previous training, and clinical exposure are associated with attitudes towards EOLC.

Nevertheless, because the study was conducted using a voluntary convenience sample from a single university, the findings should be interpreted as reflecting a specific educational context and should not be assumed to be representative of all nursing students in Spain or other countries.

Despite these limitations, the findings suggest that nursing students generally value EOLC and recognize its importance, although many do not feel adequately prepared to provide it. Educational strategies that combine theoretical instruction, clinical exposure, emotional preparation, and communication training may help bridge this gap and support the development of competence and confidence in EOLC.

### 4.1. Study Strengths

To the best of our knowledge, this is the first study to assess nursing students’ attitudes towards EOLC in Spain using a validated scale. This innovative approach not only provides a unique perspective on students’ experiences, training, and perceptions in this area but could also significantly contribute to improving undergraduate education and clinical practices related to EOLC. Furthermore, gender was collected as a variable, including the option for non-binary gender, making the study more inclusive.

### 4.2. Limitations

This study has several limitations. Regarding the study design and transferability of the findings, the cross-sectional design does not allow causal relationships to be established between the variables analyzed. In addition, the study population consisted of nursing students from a single university in southern Spain, which may limit the transferability of the findings to other nursing education settings.

With respect to the sampling strategy, the voluntary convenience sampling strategy may have introduced self-selection bias. Students with greater interest in EOLC or more favorable attitudes towards EOLC may have been more willing to participate, particularly because the topic is emotionally and ethically sensitive. Because responses were anonymous and no data were collected from students who declined participation or were absent during recruitment, non-response bias could not be formally assessed. Additionally, the sampling method used may introduce selection bias, as the sample was obtained non-randomly. The study was also conducted with students from public or affiliated institutions, which limits the ability to extrapolate the results to other age groups, academic years, or students from private or semi-private institutions.

Concerning participant characteristics and group representation, the use of gender as a variable, instead of sex, may limit direct comparability with studies that report sex-based data. In addition, the very low representation of non-binary respondents (*n* = 2) limits the interpretability of gender-based comparisons and precludes meaningful statistical inference for this subgroup. Therefore, no reliable conclusions can be drawn regarding attitudes towards EOLC among non-binary students, and the findings cannot be generalized across all gender groups. Concerning participant characteristics and subgroup comparisons, some academic years have not yet completed clinical placements and may not have faced situations requiring EOLC; hence, analysis by academic year were performed to account for differences in clinical exposure.

Regarding outcome categorization and statistical sensitivity, categorizing FATCOD-S scores may reduce statistical sensitivity and obscure within-category variation. For this reason, categories were used only for descriptive interpretation, and the continuous total score should remain the main analytical outcome.

From an analytical and psychometric perspective, the number of item-level and domain-level comparisons increases the risk of type I error. Furthermore, the conceptual domains used to summarize item-level findings were not validated as independent psychometric subscales in the present sample and should therefore be interpreted as descriptive groupings only. These results should therefore be interpreted as exploratory. In addition, although an exploratory adjusted model was added, residual confounding cannot be ruled out; therefore, adjusted associations should be interpreted cautiously and not as causal effects.

Finally, concerning self-report measures, the use of the self-report FATCOD-S questionnaire may present a limitation as responses may have been influenced by social desirability bias, particularly given the professional and ethical value attached to compassionate EOLC.

However, the questionnaire is validated for these populations and possesses good psychometric properties. In addition, perceived preparedness for EOLC was assessed using a single self-reported question rather than a validated measure of preparedness or clinical competence. Therefore, this variable should be interpreted as reflecting students’ subjective perceptions of their readiness to provide EOLC rather than their actual knowledge, skills, or competence in clinical practice. Consequently, conclusions regarding preparedness should be interpreted with caution. Although we simplified the questionnaire to reduce response time, the use of an online questionnaire could introduce acquiescence bias [48]. Nonetheless, it has been observed that bias in web-based questionnaires is not greater than that found in paper-based questionnaires [49].

## 5. Conclusions

This study shows that most nursing students display positive attitudes towards EOLC and recognize the importance of this type of care in their future professional practice. However, these favorable attitudes coexist with a relatively low perceived level of preparedness to manage end-of-life situations in clinical settings. This apparent discrepancy suggests that positive attitudes alone may not be sufficient to ensure confidence or perceived competence when caring for patients at the end of life.

The findings also suggest that many students perceive their EOLC training as insufficient. In addition, the exploratory adjusted analysis suggests that academic progression, previous EOLC training, and exposure to clinical practice may be associated with students’ attitudes and perceptions of EOLC. Taken together, these findings may suggest the potential value of educational approaches that integrate theoretical knowledge, clinical experience, communication skills, and emotional preparation related to death and the dying process.

These implications should be interpreted in light of the study’s cross-sectional design, single-university setting, voluntary convenience sample, and exploratory statistical approach. Therefore, the findings should be understood as associations within a specific educational context rather than evidence of causal effects or conclusions that can be generalized to all nursing students.

## Figures and Tables

**Table 1:** Sociodemographic and academic characteristics of the respondents and experiences related to EOLC.

			Total	
Gender	Male *n* (%)	93 (15.68)	593 (100)	
	Female *n* (%)	498 (83.98)		
	Non-binary *n* (%)	2 (0.34)		
Age (years)	median (IQR)	21.0 (20.0–22.0)		
Academic year	First year *n* (%)	135 (22.8)	593 (100)	
	Second year *n* (%)	214 (36.1)		
	Third year *n* (%)	160 (27.0)		
	Fourth year *n* (%)	84 (14.2)		
			*n*	%
Do you think EOLC is important?				
Yes			593	100.00
No			0	0.00
Do you believe that every patient has the right to receive EOLC?				
Yes			585	98.65
No			6	1.01
DK/NA			2	0.34
Have you had previous experiences with a loved one receiving EOLC?				
Yes			332	55.99
No			251	42.33
DK/NA			10	1.69
Do you experience any ethical or moral conflicts regarding EOLC?				
Yes			70	11.80
No			392	66.10
DK/NA			131	22.09
Do you believe that training in EOLC is important for nursing students?				
Yes			591	99.66
No			2	0.34
DK/NA			0	0.00
Have you received training on EOLC?				
Yes			259	43.68
No			334	56.32
DK/NA			0	0.00
If yes. do you consider the training sufficient to handle an EOLC case?				
Yes			59	9.95
No			285	48.06
DK/NA			249	41.99
Have you completed a placement in a unit where EOLC is provided?				
Yes			181	30.52
No			400	67.45
DK/NA			12	2.02
Do you feel prepared to provide care to a patient in their final days of life?				
Yes			132	22.26
No			353	59.53
DK/NA			108	18.21
Have you experienced stress related to providing EOLC during your clinical placements?				
Yes			97	16.36
No			145	24.45
DK/NA			351	59.19
Do you believe that nurses are considered in the management of EOLC?				
Yes			317	53.46
No			118	19.90
DK/NA			158	26.64
Do you believe that EOLC causes significant stress for nurses?				
Yes			350	59.02
No			52	8.77
DK/NA			191	32.21

*n*: number of students; %: percentage of students; DK/NA: don’t know, no answer.

**Table 2:** Item-level descriptive results obtained from the FATCOD-S Questionnaire.

Item	Strongly Disagree	Disagree	Neutral	Agree	Strongly Agree
	1 (*n*. %)	2 (*n*. %)	3 (*n*. %)	4 (*n*. %)	5 (*n*. %)
1. Giving nursing care to the dying person is a worthwhile learning experience	11 (1.85)	4 (0.67)	36 (6.07)	95 (16.02)	447 (75.38)
Me (Q1–Q3) = 5.0 (5.0–5.0)					
2. Death is not the worst thing that can happen to a person.	56 (9.44)	97 (16.36)	188 (31.70)	113 (19.06)	139 (23.44)
Me (Q1–Q3) = 3.0 (2.0–4.0)					
3. I would be uncomfortable talking about impending death with the dying person ^R^.	114 (19.22)	127 (21.42)	219 (36.93)	96 (16.19)	37 (6.24)
Me (Q1–Q3) = 3.0 (2.0–3.0)					
4. Nursing care should extend to the family of the dying person.	3 (0.51)	3 (0.51)	26 (4.38)	78 (13.15)	483 (81.45)
Me (Q1–Q3) = 5.0 (5.0–5.0)					
5. I would not want to be assigned to care for a dying person ^R^.	321 (54.13)	134 (22.60)	103 (17.37)	25 (4.22)	10 (1.69)
Me (Q1–Q3) = 1.0 (1.0–2.0)					
6. The nurse should not be the one to talk about death with the dying person ^R^.	360 (60.71)	142 (23.95)	74 (12.48)	13 (2.19)	4 (0.67)
Me (Q1–Q3) = 1.0 (1.0–2.0)					
7. The length of time required to give nursing care to a dying person would frustrate me ^R^.	409 (68.97)	100 (16.86)	53 (8.94)	20 (3.37)	11 (1.85)
Me (Q1–Q3) = 1.0 (1.0–2.0)					
8. I would be upset when the dying person I was caring for gave up hope of getting better ^R^.	202 (34.06)	116 (19.56)	165 (27.82)	75 (12.65)	35 (5.90)
Me (Q1–Q3) = 2.0 (1.0–3.0)					
9. It is difficult to form a close relationship with the family of the dying person ^R^.	189 (31.87)	154 (25.97)	185 (31.20)	55 (9.27)	10 (1.69)
Me (Q1–Q3) = 2.0 (1.0–3.0)					
10. There are times when death is welcomed by the dying person.	3 (0.51)	9 (1.52)	63 (10.62)	159 (26.81)	359 (60.54)
Me (Q1–Q3) = 5.0 (4.0–5.0)					
11. When a patient asks, “Nurse am I dying?,” I think it is best to change the subject to something cheerful ^R^.	318 (53.63)	151 (25.46)	103 (17.37)	17 (2.87)	4 (0.67)
Me (Q1–Q3) = 1.0 (1.0–2.0)					
12. The family should be involved in the physical care of the dying person.	1 (0.17)	19 (3.20)	63 (10.62)	149 (25.13)	361 (60.88)
Me (Q1–Q3) = 5.0 (4.0–5.0)					
13. I would hope the person I’m caring for dies when I am not present ^R^.	174 (29.34)	110 (18.55)	189 (31.87)	76 (12.82)	44 (7.42)
Me (Q1–Q3) = 3.0 (1.0–3.0)					
14. I am afraid to become friends with a dying person ^R^.	145 (24.45)	113 (19.06)	130 (21.92)	123 (20.74)	82 (13.83)
Me (Q1–Q3) = 3.0 (2.0–4.0)					
15. I would feel like running away when the person actually died ^R^.	303 (51.10)	140 (23.61)	114 (19.22)	27 (4.55)	9 (1.52)
Me (Q1–Q3) = 1.0 (1.0–3.0)					
16. Families need emotional support to accept the behavior changes of the dying person.	2 (0.34)	2 (0.34)	10 (1.69)	46 (7.76)	533 (89.88)
Me (Q1–Q3) = 5.0 (5.0–5.0)					
17. As a patient nears death, the nurse should withdraw from his or her involvement with the patient ^R^.	518 (87.35)	53 (8.94)	11 (1.85)	4 (0.67)	7 (1.18)
Me (Q1–Q3) = 1.0 (1.0–1.0)					
18. Families should be concerned about helping their dying member make the best of his or her remaining life.	2 (0.34)	6 (1.01)	20 (3.37)	80 (13.49)	485 (81.79)
Me (Q1–Q3) = 5.0 (5.0–5.0)					
19. The dying person should not be allowed to make decisions about his or her physical care ^R^.	359 (60.54)	127 (21.42)	76 (12.82)	16 (2.70)	15 (2.53)
Me (Q1–Q3) = 1.0 (1.0–2.0)					
20. Families should maintain as normal an environment as possible for their dying member.	2 (0.34)	9 (1.52)	49 (8.26)	119 (20.07)	414 (69.81)
Me (Q1–Q3) = 5.0 (4.0–5.0)					
21. It is beneficial for the dying person to verbalize his or her feelings.	1 (0.17)	5 (0.84)	23 (3.88)	55 (9.27)	509 (85.83)
Me (Q1–Q3) = 5.0 (5.0–5.0)					
22. Nursing care should extend to the family of the dying person.	1 (0.17)	5 (0.84)	41 (6.91)	106 (17.88)	440 (74.20)
Me (Q1–Q3) = 5.0 (4.0–5.0)					
23. Nurses should permit dying persons to have flexible visiting schedules.	11 (1.85)	24 (4.05)	72 (12.14)	146 (24.62)	340 (57.34)
Me (Q1–Q3) = 5.0 (4.0–5.0)					
24. The dying person and his or her family should be the in-charge decision makers.	7 (1.18)	25 (4.22)	126 (21.25)	164 (27.66)	271 (45.70)
Me (Q1–Q3) = 4.0 (3.0–5.0)					
25. Addiction to pain relieving medication should not be a concern when dealing with a dying person.	181 (30.52)	114 (19.22)	137 (23.10)	58 (9.78)	103 (17.37)
Me (Q1–Q3) = 3.0 (1.0–4.0)					
26. I would be uncomfortable if I entered the room of a terminally ill person and found him or her crying ^R^.	206 (34.74)	142 (23.95)	141 (23.78)	74 (12.48)	30 (5.06)
Me (Q1–Q3) = 2.0 (1.0–3.0)					
27 Dying persons should be given honest answers about their condition.	6 (1.01)	13 (2.19)	75 (12.65)	158 (26.64)	341 (57.50)
Me (Q1–Q3) = 5.0 (4.0–5.0)					
28. Educating families about death and dying is not a nursing responsibility ^R^.	368 (62.06)	119 (20.07)	56 (9.44)	15 (2.53)	35 (5.90)
Me (Q1–Q3) = 1.0 (1.0–2.0)					
29. Family members who stay close to a dying person often interfere with the professionals job with the patient ^R^.	60 (10.12)	115 (19.39)	262 (44.18)	103 (17.37)	53 (8.94)
Me (Q1–Q3) = 3.0 (2.0–4.0)					
30. It is possible for nurses to help patients prepare for death.	5 (0.84)	6 (1.01)	29 (4.89)	129 (21.75)	424 (71.50)
Me (Q1–Q3) = 5.0 (4.0–5.0)					

*n*: number of students; %: percentage of students; Me: median; Q: quartile; ^R^: reverse.

**Table 3:** Exploratory multivariable linear regression model for the reverse-coded FATCOD-S total score.

Predictor	Adjusted β	95% CI	*p*-Value
Age, years	−0.08	−0.32 to 0.16	0.523
Male gender vs. female	−1.95	−4.95 to 1.04	0.201
Second year vs. first year	0.94	−1.38 to 3.25	0.428
Third year vs. first year	−0.47	−3.47 to 2.52	0.758
Fourth year vs. first year	4.83	1.63 to 8.03	0.003
Previous experience with a loved one receiving EOLC: yes vs. no	1.54	−0.17 to 3.25	0.077
Clinical placement in an EOLC unit: yes vs. no	1.87	−0.32 to 4.06	0.095
Previous EOLC training: yes vs. no	2.29	0.03 to 4.55	0.047

Note. Complete-case model excluding DK/NA responses for previous experience and EOLC clinical placement and excluding the two non-binary respondents from the adjusted model because of sparse data. Female gender, first academic year, no previous loved-one EOLC experience, no EOLC unit placement and no previous EOLC training were reference categories. HC3 robust standard errors were used. β = adjusted beta coefficient; CI = confidence interval.

## Data Availability

The original contributions presented in the study are included in the article; further inquiries can be directed to the corresponding author.

## Supplementary Materials

The following supporting information can be downloaded at: https://www.mdpi.com/article/10.3390/nursrep16070233/s1, Table S1: FATCOD-S items grouped by subdimensions.

## Public Involvement Statement

No public involvement in any aspect of this research.

## Guidelines and Standards Statement

This manuscript was drafted against the Strengthening the Reporting of Observational Studies in Epidemiology (STROBE) for cross-sectional research.
