# Supplementary material for: Attitudes Towards End-of-Life Care Among Nursing Students: A Cross-Sectional Descriptive Study in a Southern European Undergraduate Nursing Program
Source: Nurs Rep. 2026 Jul 3;16(7):233. doi: 10.3390/nursrep16070233 (PMC13414523; doi:10.3390/nursrep16070233)
Supplement: Supplementary file 1 [file nursrep-16-00233-s001.zip › nursrep-4314409-supplementary.pdf]

Table S1.

FATCOD-S items grouped by subdimensions

Subdimension	Item number	Item statement
1. Emotional discomfort in caring for dying patients	3	I would not want to care for a person who is dying.
	5	I would be upset if a dying person I was caring for lost hope of getting better.
	7	I am afraid of forming a friendship with a person who is dying.
	8	When a person dies, I feel like running away.
	13	I would hope the person I'm caring for dies when I am not present.
	14	I am afraid to become friends with a dying person.
	15	I would feel like running away when the person actually died.
	26	I would be uncomfortable if I entered the room of a terminally ill person and found him or her crying.
2. Nursing care for the family	4	Nursing care for the patient's family should continue throughout the period of grief and bereavement.
	16	Families need emotional support to accept the behaviour changes of the dying person.
	22	Nursing care should extend to the family of the dying person.
3. Nurse communication and information in end-of-life care	2	Death is not the worst thing that can happen to a person. R
	6	The nurse should not be the one to talk about death with the dying person. R
	11	When a patient asks, "Nurse am I dying?" I think it is best to change the subject to something cheerful. R
	27	Dying persons should be given honest answers about their condition.
	28	Educating families about death and dying is not a nursing responsibility. R
	30	It is possible for nurses to help patients prepare for death.
4. Role of Family as caregivers	12	The family should be involved in the physical care of the dying person.
	18	Families should be concerned about helping their dying member make the best of his or her remaining life.
	20	Families should maintain as normal an environment as possible for their dying member.

5. Relational aspects of end-of-life care	1	Providing nursing care to a person who is dying is a very valuable experience.
	9	It is difficult to form a close relationship with the family of the dying person.
	10	There are times when death is welcomed by the dying person.
	17	As a patient nears death, the nurse should withdraw from his or her involvement with the patient.
	21	It is beneficial for the dying person to verbalize his or her feelings.
	29	Family members who stay close to a dying person often interfere with the professional's job with the patient.
6. Patient autonomy in end-of-life care.	19	The dying person should not be allowed to make decisions about his or her physical care.
	23	Nurses should permit dying persons to have flexible visiting schedules.
	24	The dying person and his or her family should be the in-charge decision makers.
	25	Addiction to pain relieving medication should not be a concern when dealing with a dying person.

Reverse item.
